# P03-10 I miss a normal life. It's gone on so long': A qualitative interpretation of youth's perceptions of a third national COVID-19 lockdown on their well-being and physical activity

**DOI:** 10.1093/eurpub/ckac095.046

**Published:** 2022-08-29

**Authors:** Catherine Sharp, Melitta McNarry, Liezel Hurter, Denise Hill, Gareth Stratton, Kelly Mackintosh

**Affiliations:** 1 Welsh Institute of Physical Activity, Health and Sport, Swansea University, Swansea, United Kingdom; 2 Applied Sports, Technology, Exercise and Medicine Research Centre, Swansea University, Swansea, United Kingdom

**Keywords:** Young people, Mental health, Emotional responses, COVID-19 pandemic, Human movement restrictions

## Abstract

**Background:**

Youth have experienced unprecedented restrictions during the COVID-19 pandemic. It is important to ascertain youth's perceptions of how the lockdown restrictions have impacted their well-being. Given the varying restrictions imposed in different countries, exploring the impact relative to the specific restrictions is imperative. This study investigated youth's views on the impact of a third national lockdown on their well-being and physical activity.

**Methods:**

Following informed parental consent, youth aged 8-18 years attending state schools in Wales, UK, were invited to complete an online questionnaire using an individualised link sent to the email addresses provided by parents (January 2021). A total of 4,259 survey links were issued. The questionnaire included questions on children's physical activity, mental well-being and experience of COVID-19. This study reports on free text responses from an optional two-part open-ended question on their experience during restrictions employed to manage the COVID-19 pandemic in Wales. The questions were (a) How does lockdown make you feel?, followed by (b) Why do you feel this way?. Flexible thematic analysis was employed to analyse the data and identify themes and sub-themes.

**Results:**

Valid responses were received from 1,681 youth (11.8±2.3 years; 50% girls). Most participants expressed only a negative emotional response to their lockdown experience. Whilst there were no overall sex differences in the responses, age differences were observed. Specifically, the 12-13 year-old age group reported the lowest number of negative responses, compared to 8-9 year-olds who reported the highest. Six distinct negative emotional responses were identified: sadness, anger, worry, loneliness, boredom, laziness. Nevertheless, a small cohort of participants identified positive emotional responses that focused on being happy. Mixed emotions were also reported by participants which were most prevalent amongst 16-18 year-olds and least reported in 10-11 year-olds. Finally, the inability to participate in team sports was reported negatively, however, some youth reported the additional time facilitated greater participation in exercise.

**Conclusions:**

The predominance of negative emotions highlights the significant and potentially long-lasting impact the lockdowns have had on youth's mental well-being. In addition, the findings evidence that youth associated sport participation with their mental health. Furthermore, the age differences identified highlight that youth's developmental stage, both emotionally and societally, should be considered in the recovery response to improve and reduce further deterioration in youth's mental health. This evidence should be considered when ministers evaluate the wider evidence to inform future restrictions required to manage the exit from COVID-19 and other future pandemics.

